# Gynecologic Symptoms among Hormone Receptor-Positive Breast Cancer Patients on Oral Endocrine Therapy: A Cross-Sectional Study

**DOI:** 10.3390/curroncol29030149

**Published:** 2022-03-09

**Authors:** Alexandra Moskalewicz, Amy Di Tomaso, Jacob J. Kachura, Samantha Scime, Rosane Nisenbaum, Ronita Lee, Rashida Haq, Christine Derzko, Christine Brezden-Masley

**Affiliations:** 1 St. Michael’s Hospital, 30 Bond Street, Toronto, ON M5B 1W8, Canada; a.moskalewicz@mail.utoronto.ca (A.M.); amy.ditomaso@ecrscorp.com (A.D.T.); samantha.scime@uhn.ca (S.S.); ronita.lee@unityhealth.to (R.L.); rashida.haq@unityhealth.to (R.H.); christine.derzko@unityhealth.to (C.D.); 2 Institute of Health Policy, Management and Evaluation, University of Toronto, 155 College Street Suite 425, Toronto, ON M5T 3M6, Canada; 3 Mount Sinai Hospital, 1284-600 University Avenue, Toronto, ON M5G 1X5, Canada; jkachura@bu.edu; 4 Li Ka Shing Knowledge Institute, St. Michael’s Hospital, 209 Victoria Street, Toronto, ON M5B 1T8, Canada; rosane.nisenbaum@unityhealth.to; 5 Dalla Lana School of Public Health, University of Toronto, 155 College Street 6th Floor, Toronto, ON M5T 3M7, Canada; 6 Temerty Faculty of Medicine, University of Toronto, 1 King’s College Circle, Toronto, ON M5S 1A8, Canada; 7 Lunenfeld-Tanenbaum Research Institute, Mount Sinai Hospital, 600 University Avenue, Toronto, ON M5G 1X5, Canada

**Keywords:** breast cancer, endocrine therapy, sexual dysfunction, vaginal health

## Abstract

Endocrine therapy (ET) for hormone receptor-positive (HR+) breast cancer can contribute to gynecologic symptoms (GS) that impact vaginal health, sexual function, and quality of life (QoL). A cross-sectional study was conducted at St. Michael’s Hospital in Toronto, Canada between July 2017 and June 2018 to examine the occurrence and frequency of GS among HR+ breast cancer patients on ET, patient-provider communication, female sexual dysfunction (FSD), and QoL. A Treatment Experience questionnaire was developed for this study and the Female Sexual Function Index (FSFI) and Menopause-Specific Quality of Life questionnaire (MENQOL) were also administered. Of 151 patients surveyed, 77 (51.0%) were on tamoxifen and 74 (49.0%) on an aromatase inhibitor. Most patients (84.1%, 95% confidence interval [CI] 77.3% to 89.5%) experienced at least one GS “all the time” or “often”, or one or more infections, in the past year. Only 44 (31.9%) patients reported that their oncologist had ever previously asked them about experiencing GS. The prevalence of FSD was 61.2% (95% CI 46.2% to 74.8%) among 49 sexually active patients that completed the FSFI. Symptoms captured in the MENQOL’s vasomotor domain were deemed most bothersome. Side effect management and patient-provider communication should be prioritized to optimize GS, vaginal health, and sexual function of ET users.

## 1. Introduction

Endocrine therapy (ET) is a standard treatment to target hormone receptor-positive (HR+) neoplastic growths in people with HR+ breast cancer, and is typically prescribed for five to ten consecutive years [1]. ET has significantly improved overall and disease-free survival rates but may induce a host of gynecologic symptoms (GS) which can impact vaginal health and sexual function [1,2]. In turn, the frequency and severity of these symptoms can negatively affect quality of life (QoL), and ultimately, treatment adherence [3,4]. Maintaining adherence throughout the full course of treatment is crucial to reduce the risk of cancer recurrence and mortality [5].

Oral forms of ET include tamoxifen and aromatase inhibitors (AI), such as anastrozole, letrozole and exemestane. Tamoxifen is a selective estrogen receptor modulator, whereas AIs inhibit endogenous estrogen synthesis [6]. Both mechanisms of action contribute to diminished levels of estrogen but possess varying side effect profiles [4]. Thromboembolic events and endometrial cancer, though rare, occur more commonly among patients treated with Tamoxifen, whereas AIs are associated with a higher incidence of musculoskeletal symptoms such as arthralgia and bone loss [1,4]. Menopausal symptoms such as hot flashes are common to both Tamoxifen and AI therapy [1]. In terms of GS, significant levels of vaginal dryness, dyspareunia, and decreased libido have been reported more frequently in clinical trials of post-menopausal breast cancer patients treated with anastrozole, while increased vaginal discharge, itching, and bleeding occurred more in tamoxifen users [7]. Vaginal atrophy and changes in vaginal pH are known to increase the risk of developing vaginitis or urinary tract infections (UTI) during ET [8]. In a cross-sectional study by Chin et al., 63% of post-menopausal patients on ET reported experiencing some degree of urogenital symptoms at the time of survey, with vaginal dryness identified as the most commonly reported symptom [9].

Hormonal changes underlying the GS that some patients experience while on ET can contribute to female sexual dysfunction (FSD) [8]. FSD is a multi-faceted issue in breast cancer survivors that is both physiological and psychosocial in nature, and typically encompasses difficulties with desire, arousal, orgasm, lubrication, and dyspareunia [8,10]. In a meta-analysis of studies which administered the Female Sexual Function Index (FSFI), the pooled prevalence of FSD in women with all types of breast cancer was 73.4% (95% confidence interval [CI] 64.0% to 82.8%, *I*^2^ = 98.8%) [10]. Aside from ET, undergoing chemotherapy, ovarian function suppression therapy, or an oophorectomy can contribute to treatment-induced menopause and diminished sexual function. Furthermore, breast surgery itself may result in negative changes to nipple sensitivity and stimulation, as well as body image [3,8].

Vaginal health and sexual function are topics that remain widely unaddressed both during and after breast cancer treatment [3,11,12]. Communication around sexual concerns have been described as infrequent and reluctant from both patient and provider perspectives [12,13]. Despite sexual activity, maintaining vaginal health is important for breast cancer survivors of any age to ensure daily comfort and prevent infection [11].

To date, few cross-sectional studies have focused primarily on GS when investigating side effects of ET and patient-provider communication among HR+ breast cancer patients [9,11,14,15,16]. We conducted a cross-sectional study as part of an internal needs assessment at St. Michael’s Hospital (SMH) in Toronto, Ontario, Canada, with the primary objective of examining the occurrence and frequency of GS in HR+ breast cancer patients on oral ET. Secondary objectives were to examine patient-provider communication around GS, FSD, and QoL. As exploratory objectives, GS, FSD, and QoL were compared by ET type (tamoxifen versus AI).

## 2. Materials and Methods

### 2.1. Participant Recruitment

Recruitment for this cross-sectional study was conducted between July 2017 and June 2018 in the outpatient oncology clinic at SMH. Potentially eligible patients were identified from the medical oncologists’ clinic rosters and a convenience sampling strategy was used [17]. Patients were approached to participate during their appointment and three paper-based questionnaires were distributed to complete within the clinic on the day of consent. Women with any stage HR+ breast cancer and any menopausal status were eligible to participate if they had a treatment history with tamoxifen or an AI (anastrozole, letrozole or exemestane) for six or more consecutive months prior to study participation. Patients receiving ovarian function suppression therapy in combination with one of the aforementioned ET options were also permitted to participate. Those with a concurrent gynecologic malignancy or condition were ineligible. Research ethics board approval was obtained at SMH and all participants provided informed consent.

### 2.2. Measures and Data Collection

Firstly, we developed a treatment experience questionnaire for use in this study to obtain self-reported information on a series of GS, sexual history, patient-provider communication, and patient needs in relation to their side effects (Appendix A). It was not pilot-tested prior to use, nor were its psychometric properties examined. The frequency of the following GS were assessed: vaginal dryness, vaginal discharge, vaginal bleeding, vaginal itchiness, hot flashes/insomnia, decreased sex drive, feelings of depression, UTIs, and yeast infections/vaginitis. Response options to describe each symptom’s frequency in the past year were: all the time, often (at least once a week), rarely (a few times a year) or never. For UTIs and yeast infections/vaginitis, patients were asked to write the number of each infection they have experienced in the past year. These responses were then grouped into None, 1 to 2, 3 to 4, and 5 or more. With regard to sexual history, patients were asked if they were currently sexually active, had a sexual partner, and if they experienced pain during intercourse or masturbation. To examine patient-provider communication, patients indicated if their oncologist had ever asked them about experiencing any of the aforementioned GS (i.e., sexual and/or vaginal health), and if they are comfortable talking about their sexual and/or vaginal health with their oncologist. To assess needs, patients were asked to keep their current side effects in mind and select as many of the following statements that applied to them: “I am interested in seeing a physician specialist concerning my side effects”, “I am open to potential treatment for my side effects”, “I want to know what my options are regarding how to best address my side effects”, “My side effects aren’t severe enough for me to want treatment”, and “Other”. Open-ended responses were permitted under the “Other” response option, which were subsequently coded to identify common themes using a thematic analysis approach [18].

The FSFI was used to describe sexual function and determine the prevalence of FSD [19]. The FSFI uses a one-month recall and contains 19 items across desire, arousal, lubrication, orgasm, satisfaction, and pain domains. Domains are scored individually and a total score can be obtained (range 2.0–36.0). A higher score indicates higher sexual function [19]. A diagnostic cut-off score of ≤26.55 has been established to suggest FSD at the time of survey [20]. The FSFI’s validity, reliability and cut-off score have been previously described [19,20,21]. FSFI validation studies in patients with breast cancer and other malignancies have demonstrated psychometric soundness, though only in sexually active participants [22,23].

The Menopause-Specific Quality of Life questionnaire (MENQOL) was used to examine QoL [24]. The one-week recall version evaluates 29 symptoms across vasomotor, psychosocial, physical, and sexual domains. Participants indicate if they have experienced a symptom and rate how bothersome it is. After converting scores to a scale from one to eight, a domain score of one indicates no symptoms were experienced, and eight indicates being extremely bothered by the symptoms captured in the domain [24]. The total score equates to the average of the domain scores [24]. The MENQOL’s psychometric properties have been demonstrated as acceptable to assess treatment-induced menopausal symptoms in women with breast cancer [25].

Patient characteristics and cancer-related medical history were collected through electronic medical record (EMR) review. This included age and menopausal status at breast cancer diagnosis, cancer treatment and surgical history, as well as ET type and age at time of survey. Oncology clinic transcriptions were reviewed since ET was first prescribed and prior to the patient’s survey date for any instance of ET non-adherence. We defined this as any lapse in adherence attributed to side effects of any nature that were noted by the oncologist, rather than any mutually agreed upon pauses from ET.

### 2.3. Statistical Analysis

Patient characteristics, treatment experience questionnaire items, FSFI scores, and MENQOL scores were reported descriptively as counts and percentages or means and standard deviations (SD). The Chi-square or Fisher’s exact test was used to examine differences in the frequency of GS, patient-provider communication-related questionnaire items, and FSD by binary patient characteristics or ET type. A binomial proportion and exact (Clopper-Pearson) 95% confidence interval was calculated for the proportion of sexually active patients with FSD, and the proportion of patients who experienced at least one GS either “all the time” or “often”, or one or more infections, in the past year.

Domain and total instrument scores for the FSFI and MENQOL were computed using the appropriate scoring manuals. We followed the recommendations of Baser, Li, and Carter and omitted patients who were deemed not sexually active from scoring of the FSFI to avoid downward-biased domain and total scores, which can inflate the prevalence of FSD [22]. Only patients who answered “yes” to the question: “Are you currently sexually active?” in the treatment experience questionnaire were included in FSFI scoring. We then excluded any patient from scoring who indicated “no sexual activity” within any of the FSFI’s questions 3–14, or “did not attempt intercourse” in questions 17–19, or skipped eight or more responses [22]. The prevalence of FSD was reported as a count and percentage, and represented the number of sexually active patients who fully completed the FSFI and obtained a total score of ≤26.55 [20]. The Wilcoxon rank sum test was used to test for differences in FSFI and MENQOL domain and total scores by ET type. A complete case analysis was used to report individual questionnaire items, as well as FSFI and MENQOL domain and total scores. Analyses were performed using SAS 9.4 (SAS Institute, Cary, NC, USA). Statistical tests were two-tailed and *p*-values less than 0.05 were used to define statistical significance.

## 3. Results

**Figure 1:**
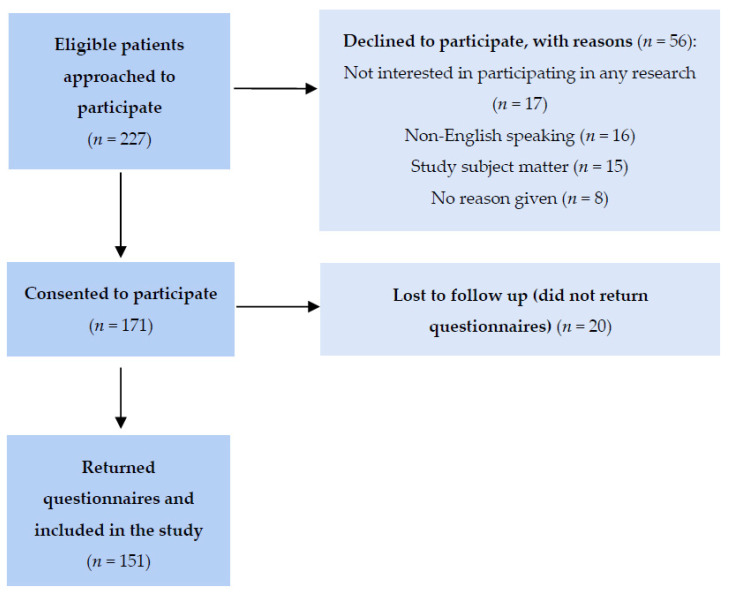
Flow diagram of study participant selection.

A total of 227 eligible patients were approached, to which 171 consented to participate and 56 declined for various reasons (Figure 1). Twenty patients did not return the questionnaires and were considered lost to follow-up. A total of 151 patients returned the questionnaires and comprised the study sample (88.3% response rate).

### 3.1. Patient Characteristics

At the time of the survey, the mean age was 56.2 ± 10.3 years (Table 1). About half (77/151, 51.0%) of breast cancer patients were taking tamoxifen and the other half (74/151, 49.0%) an AI. Seventy-one (48.0%) were sexually active and ninety-six (64.4%) indicated having a sexual partner at the time of survey. Forty-five (35.4%) patients reported pain during intercourse or masturbation. One or more lapses in ET adherence attributed to side effects were noted in the EMR by the oncologist for seventeen (11.3%) patients since first being prescribed ET and prior to their survey date.

### 3.2. Occurrence and Frequency of GS

Table 2 displays a series of GS and how frequently they occurred in breast cancer patients on ET in the year prior to their survey date. Hot flashes (44.7%) and a decreased sex drive (37.7%) were the symptoms most commonly experienced “all the time”. “Never” was the most commonly selected symptom frequency option for vaginal dryness (32.4%), feeling depressed (34.1%), vaginal discharge (46.8%), vaginal itchiness (51.8%) and vaginal bleeding (82.5%). Most patients reported zero UTIs (85.9%) and zero yeast infections/vaginitis (84.6%). Overall, 127 patients (84.1%, exact 95% CI 77.3% to 89.5%) reported experiencing at least one of the aforementioned GS either “all the time” or “often”, or one or more infections, in the past year. The frequency of GS by ET type is presented in Table S1. A statistically significant association between the frequency of the following symptoms and ET type was observed: vaginal dryness (*p* = 0.021), vaginal discharge (*p* = < 0.001), hot flashes/insomnia (*p* = 0.027), and decreased sex drive (*p* = 0.012).

### 3.3. Patient-Provider Communication and Patient Needs around GS

Only 44 (31.9%) breast cancer patients reported being previously asked by their oncologist about experiencing any GS, though most patients (123, 87.9%) reported being comfortable to discuss their sexual and/or vaginal health with their oncologist. No statistically significant differences were observed with regard to whether the patient’s oncologist had ever asked them about GS (yes/no) by patient age ≥ 50 years compared to <50 years at time of survey (*p* = 0.238), ET type (*p* = 0.641), being sexually active (*p* = 0.059), or having a sexual partner (*p* = 0.302).

When prompted to indicate which statements applied to their current side effects, almost half (68, 45.0%) of patients indicated that theirs were not severe enough to want treatment, 34 (22.5%) indicated wanting to know what their options were to best address them, 21 (13.9%) would be interested in seeing a physician specialist and 28 (18.5%) would be open to potential treatment. Nineteen (12.6%) patients selected ‘Other’ and provided an open-ended response. After grouping into themes, eleven patients indicated that they were not currently experiencing any concerning gynecologic side effects, six were no longer interested in sex, and two emphasized the severity of their current side effects.

### 3.4. Sexual Function

One or more FSFI domain scores could be calculated for 55 sexually active breast cancer patients (Table 3). The desire and arousal domains incurred the lowest mean scores of 2.9 ± 1.1 and 3.8 ± 1.2, respectively. A total FSFI score could be calculated for 49 of the 55 patients, with a resulting mean of 24.2 ± 6.3. The prevalence of FSD (total score of ≤26.55) at the time of survey among these 49 patients was 61.2% (exact 95% CI 46.2% to 74.8%). Of the 30 patients with FSD, 20 (66.7%) were ≥50 years, 15 (50.0%) were on tamoxifen, and 15 (50.0%) an AI. There was no statistically significant difference in FSD status by ET type (*p* = 0.139) or age ≥50 vs. <50 years (*p* = 0.326). When FSFI scores were compared by ET type, a statistically significant association was not observed for any domain (Table S2).

### 3.5. QoL

MENQOL domain and total scores are reported in Table 4. The vasomotor domain incurred the highest mean score of 4.1 ± 2.3, indicating that symptoms captured within this domain, such as hot flashes, were deemed most bothersome. A total score could be calculated for 98 breast cancer patients who completed all MENQOL domains, which resulted in a mean total score of 3.9 ± 1.7. Of these 98 patients, 50 (51.0%) were on tamoxifen and 48 (49.0%) an AI. No significant differences in MENQOL domains or total score were observed by ET type (Table S3).

## 4. Discussion

In this cross-sectional study, the majority of breast cancer patients on oral ET reported experiencing one or more GS “all the time” or “often” in the year prior to their survey date. A lack of communication was identified between patients and their oncologists around GS, though most patients expressed being comfortable to discuss them. FSD was a prevalent issue, identified in over half of sexually active patients who completed the FSFI. Vasomotor and sexual-related symptoms were most bothersome to patients, as they incurred the highest mean scores within the MENQOL questionnaire.

The frequency of self-reported GS appears to be high but variable across studies of ET users. This could be partially attributed to differences in study design and methodology, questionnaires employed and associated recall periods, as well as the clinical characteristics and treatment history of the study samples. Hot flashes/insomnia was the most frequently reported GS in our study, which is consistent with hot flashes and vasomotor symptoms in general being identified as one of the most commonly reported side effects across both tamoxifen and AI users in clinical trials [4]. In comparison with other single-center cross-sectional research, Chin et al., surveyed post-menopausal patients on ET, with 48% (*n* = 121) of patients self-reporting mild, moderate, or severe vaginal dryness, though a much shorter recall period of seven days was used to assess side effects [9]. The low frequency of vaginal itchiness, bleeding, discharge and UTIs we observed is also comparable with Chin et al. [9]. Our results around the frequency of experiencing a decreased sex drive (in the past year) appear higher than Lin et al., who surveyed estrogen receptor-positive breast cancer patients on ET and reported decreased libido in 24% of ET users in the past seven days [14].

The duration of ET may also play a role in the frequency and severity of GS. In the Arimidex, Tamoxifen, Alone or in Combination (ATAC) trial, endocrine symptoms worsened at three months from baseline, then stabilized or slightly improved [7]. In the Tamoxifen and Exemestane Trial (TEXT) and the Suppression of Ovarian Function Trial (SOFT), sexual problems in pre-menopausal breast cancer patients increased at six months from baseline and persisted for up to two years [26]. Our eligibility criteria attempted to account for this effect by surveying patients with an ET treatment history of six or more months.

The discord we observed between the lack of discussion but willingness to converse among patients and their oncologist around GS is consistent with previous literature in female breast cancer survivors [27,28]. Some evidence suggests that oncology healthcare providers may rely on various characteristics such as age or marital status to make assumptions about sexual activity before initiating discussions around sexual health [12]. However, we did not find any factors (age at survey, age at diagnosis or having a sexual partner) to be statistically significantly associated with whether an oncologist had ever inquired about GS. Almost one-quarter of patients in our study wanted to know more about options to best address their side effects, indicating that they may be unaware of available treatment options and may have possibly accepted them as a new standard of life [27]. Breast cancer patients have expressed uncertainty in qualitative studies as to whether their symptoms and side effects were attributable to their ET, menopause and aging, comorbidities, or even cancer recurrence [29,30]. There are several guideline-recommended first-line, nonhormonal options for GS accessible over-the-counter that oncologists can suggest to their breast cancer patients before assessing if additional intervention or support would be beneficial, such as vaginal moisturizers and lubricants to target vaginal dryness and dyspareunia [12,31].

FSD, as classified by the FSFI, was identified in 61.2% of the sexually active patients within our study sample. A cross-sectional study which similarly applied the FSFI solely in sexually active breast cancer patients on ET and used the cut-off score of ≤26.55 reported FSD in 63% (142/227) of their study sample [32]. When comparing FSFI domain scores by ET type, we observed no significant differences, which is inconsistent with previous observational studies evaluating the symptoms covered by these domains [16]. In a cross-sectional study with age-matched controls by Baumgart et al., rates of insufficient lubrication, dissatisfaction with sex life, and reduced interest in sex (in the last five years) were significantly higher in AI users than those on tamoxifen [16]. However, Baumgart et al., used survey-based estimates to assess the above-mentioned issues related to sexual dysfunction while we used the FSFI.

Through retrospective review of transcriptions in our institution’s EMR, oncologists noted one or more lapses in adherence due to side effects in 11.3% of patients prior to their survey date. Rates of non-adherence have been noted elsewhere to be as high as 30–50% of breast cancer patients who initiate ET, with treatment-related symptoms being one of the most common factors contributing to decreased adherence or discontinuation [1,33]. In terms of reporting measures for ET adherence, Font et al., found that physicians reported higher rates of adherence in their patients, while patient self-report or linkage to prescription refill records resulted in lower estimates of adherence [34]. This may suggest that our estimate, produced through chart review, may be an underestimation and also lacks detailed reasoning behind non-adherence than if patients were surveyed about adherence with a validated measure.

This study contributes to the limited evidence base of observational studies which examine the occurrence and frequency of GS secondary to ET in HR+ breast cancer patients, as well as patient-provider communication around these symptoms. However, several limitations are worth mentioning. Our results may not be entirely generalizable to all patients on ET as this was a single-center study which utilized convenience sampling for recruitment, as opposed to consecutive or random sampling. A sample size calculation was not conducted, which led to our study being potentially underpowered for the exploratory analyses by ET type. Additionally, some socio-demographic and clinical variables that were not collected could have acted as confounding variables, such as marital status, history of radiation therapy or hysterectomy, clinical indication of premature ovarian failure, comorbidities, body mass index, or concomitant medications. Menopausal status and mean duration of ET use at the time of survey is unknown, though these factors could have influenced the occurrence and frequency of GS reported. A control group was not included for formal comparison to examine any associations of symptoms with ET, and we cannot conclude whether any self-reported symptoms, or FSD, were truly attributable to ET or existed prior to ET initiation. Recall bias could have influenced patient responses, as the three questionnaires administered contained differing recall periods. Patients who agreed to participate in our study may have been experiencing more negative or heightened effects from ET and/or may have been more comfortable being surveyed about their vaginal health and sexual function. Besides the nature of the subject matter, it is possible that the set of questionnaires were perceived as too long or burdensome by patients, contributing to the amount of missing data observed. As one strategy to improve recruitment efforts and reduce missing responses, researchers conducting future studies to examine GS in ET users may wish to engage with patient partners when creating new data collection measures and selecting existing ones for use.

## 5. Conclusions

GS were widespread at the time of survey among HR+ breast cancer patients on oral ET, as was FSD in sexually active patients, while patient-provider communication around these issues appeared to be sparse. As prolonged treatment with ET beyond five years is increasingly being advised, ensuring that patients have adequate symptom management strategies and open communication around possible treatment-related effects with their health care team are essential strategies to ensure needs are addressed and that treatment adherence is sustained. Continued efforts to bring awareness to vaginal and sexual health concerns in clinical practice will impact its normalcy as a topic of discussion during breast cancer treatment and its relevance throughout survivorship.

## Figures and Tables

**Table 1:** Patient characteristics.

	All Patients
	*n* = 151
Age at diagnosis, years (mean ± SD)	52.2 ± 10.2
<50	66 (43.7%)
≥50	85 (56.3%)
Tumor characteristics	
HR+, HER2-negative	123 (81.5%)
HR+, HER2-positive	28 (18.5%)
Menopausal status at diagnosis	
Pre-menopausal	68 (45.0%)
Peri-menopausal	11 (7.3%)
Post-menopausal	72 (47.7%)
History of chemotherapy	87 (57.6%)
Breast cancer surgery	
Lumpectomy	96 (63.6%)
Mastectomy	48 (31.8%)
None	7 (4.6%)
History of breast reconstruction	38 (25.2%)
Prophylactic oophorectomy to initiate ET	17 (11.3%)
Use of hormone replacement therapy prior to breast cancer diagnosis	19 (12.6%)
Age at time of survey, years (mean ± SD)	56.2 ± 10.3
<50	38 (25.2%)
≥50	113 (74.8%)
Metastatic cancer at time of survey	18 (11.9%)
Current ET at time of survey	
Tamoxifen	77 (51.0%)
Anastrozole	33 (21.9%)
Letrozole	23 (15.2%)
Exemestane	18 (11.9%)
Concurrent ovarian function suppression therapy with ET at time of survey	9 (6.0%)
Lapse in ET adherence ever noted in EMR	17 (11.3%)

Note: Data for each characteristic are presented as frequency (percent) unless otherwise specified. Abbreviations: HR+, hormone receptor-positive; HER2, human epidermal growth factor receptor 2; EMR, electronic medical record; ET, endocrine therapy.

**Table 2:** Frequency of self-reported gynecologic symptoms (GS) in the past year.

Symptom	*n* (%) ^1^	All the Time	Often ^2^	Rarely ^3^	Never
Vaginal dryness	142	42 (29.6)	14 (9.9)	40 (28.2)	46 (32.4)
Vaginal discharge	141	17 (12.1)	27 (19.2)	31 (22.0)	66 (46.8)
Vaginal bleeding	137	2 (1.5)	1 (0.7)	21 (15.3)	113 (82.5)
Vaginal itchiness	137	5 (3.7)	16 (11.7)	45 (32.9)	71 (51.8)
Hot flashes/insomnia	141	63 (44.7)	32 (22.7)	30 (21.3)	16 (11.4)
Decreased sex drive	130	49 (37.7)	32 (24.6)	25 (19.2)	24 (18.5)
Feel depressed	132	14 (10.6)	35 (26.5)	38 (28.8)	45 (34.1)
		**1 to 2**	**3 to 4**	**5 or more**	**None**
Urinary tract infection (UTI)	135	12 (8.9)	4 (3.0)	3 (2.2)	116 (85.9)
Yeast infection/vaginitis	130	14 (10.8)	5 (3.9)	1 (0.8)	110 (84.6)

^1^*n* represents the number of breast cancer patients who responded to each question, of the total sample size of 151 patients. ^2^ At least once a week. ^3^ A few times a year.

**Table 3:** FSFI domain and total scores.

FSFI Domain	*n* ^1^	Score (Mean ± SD)
Desire	55	2.9 ± 1.1
Arousal	54	3.8 ± 1.2
Lubrication	53	4.4 ± 1.4
Orgasm	55	4.4 ± 1.3
Satisfaction	52	4.5 ± 1.3
Pain	52	4.5 ± 1.6
Total score	49	24.2 ± 6.3

Abbreviations: FSFI (Female Sexual Function Index). ^1^ *n* indicates the number of breast cancer patients who completed the FSFI domain, of the total sample size of 55 sexually active patients who had one or more FSFI domain scored.

**Table 4:** MENQOL domain and total scores.

MENQOL Domain	*n* ^1^	Score (Mean ± SD)
Vasomotor	129	4.1 ± 2.3
Psychosocial	122	3.6 ± 1.8
Physical	114	3.7 ± 1.6
Sexual	119	3.9 ± 2.4
Total score	98	3.9 ± 1.7

Abbreviations: MENQOL (Menopause-Specific Quality of Life questionnaire). ^1^ *n* indicates the number of breast cancer patients who completed all items within a specific MENQOL domain for it to be scored, of the total sample size of 151 patients.

## Data Availability

The data presented in this study are available on request from the corresponding author. The data are not publicly available due to patient privacy.

## Supplementary Materials

The following supporting information can be downloaded at: https://www.mdpi.com/article/10.3390/curroncol29030149/s1. Table S1: Frequency of self-reported GS in the past year by ET type; Table S2: FSFI domain and total scores by ET type; Table S3: MENQOL domain and total scores by ET type.

## Appendix A. Treatment Experience Questionnaire

We are hoping to learn more about the quality of life, vaginal health and sexual health of our patients receiving endocrine therapy for their breast cancer. Your responses will help us better understand if and how endocrine therapy impacts the sexual health and quality of life of our patients. We thank you very much for your time in participating.

1. How old were you when you were diagnosed with breast cancer?
  ______________________
2. At the time of diagnosis, what endocrine therapy did your doctor prescribe to you?
  a. Tamoxifen
  b. Aromatase Inhibitors (anastrozole/letrozole/exemestane)
  c. Ovarian function suppression (goserelin acetate/oophorectomy)
  d. I am not sure
3. Has your endocrine therapy changed since then?
  a. Yes
  b. No
  c. I am not sure
4. If YES, why did the treatment change?
  a. Your oncologist recommended the change
  b. You experienced side effects
  c. Other (please be specific) ______________________
5. At diagnosis, were you (please circle one):
  a. Pre-menopausal, peri-menopausal, post-menopausal, I am not sure.
6. Did your period stop after starting cancer treatment?
  a. Yes
  b. No
  c. I am not sure
7. To the best of your memory, when was your last period (month and year)?
  ______________________
8. With regards to your menstrual history:
  a. When did you get your first period (age in years)? ______________________
  b. What was the average length of your menstrual cycle (in days)?______________________
  c. How many days did your period usually last, on average?______________________
9. With regards to your pregnancy history (if not applicable, skip to question 10):
  a. How many times have you been pregnant?______________________
  b. How many children have you delivered? ______________________
  c. How many were delivered vaginally? ______________________
  d. How many of them were delivered via C-section? ______________________
  e. How many pregnancies have you lost (i.e., miscarriage, abortion) if applicable? ______________________

Please answer these questions to the best of your ability. Your responses will help us better understand if and how endocrine therapy impacts the sexual health and quality of life of our patients. Your responses will be de-identified and kept completely confidential.

10. In the past year, how often have you experienced the following?

	**All the Time**	**Often (at Least Once a Week)**	**Rarely (a Few Times)**	**Never**
Vaginal dryness				
Vaginal discharge				
Vaginal bleeding				
Vaginal itchiness				
Urinary tract infections (UTIs) (please write the number of UTIs you’ve had in the past year)				
Yeast infections/vaginitis (please write the number of yeast infections/vaginitis you’ve had in the past year)				
Hot flashes/insomnia				
Decreased sex drive				
Feel depressed				

11. Are you currently sexually active?
  a. Yes
  b. No
12. Do you have a sexual partner?
  a. Yes
  b. No
13. Do you experience pain during intercourse or masturbation?
  a. Yes
  b. No
14. Has your oncologist asked you about any of the previously mentioned side effects? (i.e., sexual and/or vaginal health)?
  a. Yes (continue to the next question)
  b. No (skip to question 17)
15. If YES (i.e., you selected option A for question 14), what happened next (check all that apply)?
  a. Your doctor told you about different treatment options that could be offered to you
  b. Your doctor advised you against local hormone therapy (i.e., vaginal estrogen creams)
  c. Your doctor referred you to a specialist/gynecologist
  d. Your symptoms are mild and you did not want to take action
  e. Other (please specify)
    __________________________________________________________________________________________________________________________________________________________________________________
16. If you were referred to a specialist/gynecologist (i.e., you selected option C for question 15), what happened next (check all that apply)?
  a. It was recommended that you stop endocrine therapy (stopped Tamoxifen or aromatase inhibitors)
  b. It was recommended that you change cancer treatment
  c. You were advised against local hormone therapy
  d. You received supportive medication for your side effects (e.g., vaginal creams, local hormone therapy)
  e. Other (please specify)
    __________________________________________________________________________________________________________________________________________________________________________________
17. If your oncologist did NOT ask you about these side effects (i.e., you selected option B for question 14), did you bring it up with them?
  a. Yes
  b. No
18. If YES (i.e., you selected option A for question 17), what happened next?
  a. Your doctor told you about different treatment options that could be offered to you
  b. Your doctor advised you against local hormone therapy (i.e., vaginal estrogen creams)
  c. Your doctor referred you to a specialist/gynecologist
  d. Your symptoms are mild and you did not want to take action
  e. Other (please specify)
    __________________________________________________________________________________________________________________________________________________________________________________
19. If NO (i.e., you selected option B for question 17), why didn’t you bring it up?
  a. Embarrassment (you find it difficult/awkward talking about your sexual and/or vaginal health)
  b. There was no opportunity to bring it up
  c. The side effects are not severe enough for you to want treatment
  d. Other (please specify)
    __________________________________________________________________________________________________________________________________________________________________________________
20. If you were referred to a specialist/gynecologist (i.e., you selected option C for question 18), what happened next (check all that apply)?
  a. It was recommended that you stop endocrine therapy (stopped Tamoxifen or aromatase inhibitors)
  b. It was recommended that you change cancer treatment
  c. You were advised against local hormone therapy
  d. You received supportive medication for your side effects (e.g., vaginal creams, local hormone therapy)
  e. Other (please specify)
    __________________________________________________________________________________________________________________________________________________________________________________
21. If applicable, did your side effects improve with the change in endocrine therapy or additional supportive medication?
  a. Yes
  b. No
22. If NO (i.e., you selected option B for question 21), what happened next?
  __________________________________________________________________________________________________________________________________________________________________________________
23. Are you comfortable talking about your sexual health and/or vaginal health with your oncologist?
  a. Yes
  b. No
24. If NO (i.e., you selected option B for question 23), why are you uncomfortable speaking to your oncologist about this topic?
  __________________________________________________________________________________________________________________________________________________________________________________
25. With your current side effects in mind, check all that apply:
  a. I am interested in seeing a physician specialist concerning my side effects
  b. I am open to potential treatment for my side effects
  c. I want to know what my options are regarding how to best address my side effects
  d. My side effects aren’t severe enough for me to want treatment
  e. Other (please specify)
    __________________________________________________________________________________________________________________________________________________________________________________
