# Supplementary material for: Gynecologic Symptoms among Hormone Receptor-Positive Breast Cancer Patients on Oral Endocrine Therapy: A Cross-Sectional Study
Source: Curr Oncol. 2022 Mar 9;29(3):1813–27. doi: 10.3390/curroncol29030149 (PMC8947613; doi:10.3390/curroncol29030149)
Supplement: Supplementary file 1 [file curroncol-29-00149-s001.zip › curroncol-1587081-supplementary.pdf]

Current Oncology

MDPI

Article

Gynecologic Symptoms among Hormone Receptor-Positive Breast Cancer Patients on Oral Endocrine Therapy: A Cross-Sectional Study

Alexandra Moskalewicz, Amy Di Tomaso, Jacob J. Kachura, Samantha Scime, Rosane Nisenbaum, Ronita Lee, Rashida Haq, Christine Derzko and Christine Brezden-Masley

Table S1: Frequency of self-reported gynecologic symptoms (GS) in the past year by endocrine therapy (ET) type.

	Tamoxifen users (*n*, %)					AI users (*n*, %)					
**Symptom**	***n*** [1]	**All the time**	**Often** [2]	**Rarely** [3]	**Never**	***n*** [1]	**All the time**	**Often** [2]	**Rarely** [3]	**Never**	***p*-value**
**Vaginal dryness**	73	14 (19.2)	10 (13.7)	25 (34.2)	24 (32.9)	69	28 (40.6)	4 (5.8)	15 (21.7)	22 (31.9)	0.021
**Vaginal discharge**	75	15 (20.0)	21 (28.0)	16 (21.3)	23 (30.7)	66	2 (3.0)	6 (9.1)	15 (22.7)	43 (65.2)	<.001
**Vaginal bleeding**	71	1 (1.4)	1 (1.4)	14 (19.7)	55 (77.5)	66	1 (1.5)	0 (0.0)	7 (10.6)	58 (87.9)	0.266
**Vaginal itchiness**	72	2 (2.8)	10 (13.9)	23 (31.9)	37 (51.4)	65	3 (4.6)	6 (9.2)	22 (33.8)	34 (52.3)	0.821
**Hot flashes/insomnia**	75	34 (45.3)	14 (18.7)	22 (29.3)	5 (6.7)	66	29 (43.9)	18 (27.3)	8 (12.1)	11 (16.7)	0.027
**Decreased sex drive**	70	18 (25.7)	23 (32.9)	16 (22.9)	13 (18.6)	60	31 (51.7)	9 (15.0)	9 (15.0)	11 (18.3)	0.012
**Feel depressed**	69	8 (11.6)	15 (21.7)	25 (36.2)	21 (30.4)	63	6 (9.5)	20 (31.7)	13 (20.6)	24 (38.1)	0.193
		**1 to 2**	**3 to 4**	**5 or more**	**None**		**1 to 2**	**3 to 4**	**5 or more**	**None**	
**Urinary tract infection (UTI)**	69	5 (7.2)	2 (2.9)	0 (0.0)	62 (89.9)	66	7 (10.6)	2 (3.0)	3 (4.5)	54 (81.8)	0.338
**Yeast infection/vaginitis**	69	8 (11.6)	5 (7.2)	1 (1.4)	55 (79.7)	61	6 (9.8)	0 (0.0)	0 (0.0)	55 (90.2)	0.087

Abbreviations: AI (Aromatase inhibitor). [1] *n* represents the number of breast cancer patients who responded to each question, of the total sample size of 151 patients. [2] At least once a week. [3] A few times a year.

Table S2. FSFI domain and total scores by ET type.

	Tamoxifen users		AI users		
**FSFI domain**	***n*** [1]	**Score (mean ± SD)**	***n*** [1]	**Score (mean ± SD)**	***p*-value**
Desire	35	3.02 ± 1.03	20	2.64 ± 1.24	0.188
Arousal	34	3.91 ± 1.17	20	3.50 ± 1.26	0.286
Lubrication	33	4.61 ± 1.50	20	4.13 ± 1.22	0.090
Orgasm	35	4.49 ± 1.11	20	4.18 ± 1.66	0.661
Satisfaction	32	4.68 ± 1.21	20	4.28 ± 1.44	0.308
Pain	32	4.63 ± 1.52	20	4.42 ± 1.81	1.000
Total score	29	24.89 ± 6.24	20	23.14 ± 6.51	0.334

Abbreviations: FSFI (Female Sexual Function Index). [1] *n* indicates the number of breast cancer patients who completed the FSFI domain, of the total sample size of 55 sexually active patients who had one or more FSFI domain scored.

Table S3. MENQOL domain and total scores by ET type.

	Tamoxifen users		AI users		
**MENQOL domain**	***n*** [1]	**Score (mean ± SD)**	***n*** [1]	**Score (mean ± SD)**	***p*-value**
Vasomotor	67	4.20 ± 2.32	62	4.03 ± 2.35	0.692
Psychosocial	62	3.50 ± 1.74	60	3.73 ± 1.84	0.525
Physical	56	3.56 ± 1.44	58	3.90 ± 1.76	0.357
Sexual	63	3.57 ± 2.11	56	4.26 ± 2.63	0.185
Total score	50	3.69 ± 1.48	48	4.06 ± 1.85	0.338

Abbreviations: MENQOL (Menopause-Specific Quality of Life questionnaire). [1] *n* indicates the number of breast cancer patients who completed all items within a specific MENQOL domain for it to be scored, of the total sample size of 151 patients.
